# Environmental predictability drives different routes to adaptation

**DOI:** 10.1093/evlett/qraf052

**Published:** 2025-12-31

**Authors:** Anna C Vinton, Ben Ibbott, Jinlin Chen, Samuel J L Gascoigne, Dominique Baptiste, Tim Coulson

**Affiliations:** Department of Biology, University of Oxford, Oxford, United Kingdom; Department of Marine and Environmental Sciences, University of Southern California, Los Angeles, United States; Wrigley Institute for Environment and Sustainability, University of Southern California, Los Angeles, United States; College of Arts & Sciences, University of Maine at Presque Isle, Presque Isle, United States; Department of Biology, University of Oxford, Oxford, United Kingdom; Department of Biology, University of Oxford, Oxford, United Kingdom; School of Life Sciences, Nanjing University, Nanjing, China; Department of Biology, University of Oxford, Oxford, United Kingdom; School of Biological Sciences, University of Aberdeen, Aberdeen, United Kingdom; Department of Biology, University of Oxford, Oxford, United Kingdom; Department of Biology, University of Oxford, Oxford, United Kingdom

**Keywords:** environmental predictability, phenotypic plasticity, thermal adaptation, *Drosophila melanogaster*, life-history evolution, temperature variation

## Abstract

Climate change is altering thermal environments, yet we know little about how environmental predictability shapes species' adaptive responses. Different species may rely on plasticity or evolution to survive environmental change, but how these strategies depend on environmental predictability remains unclear. Experimental evidence that distinguishes between plastic and evolutionary responses to different patterns of environmental variability has been lacking. Here, we present the first experimental demonstration that compares adaptive responses to predictable versus unpredictable thermal variation, disentangling plastic from evolutionary changes. Using *Drosophila melanogaster* populations evolved for 11 generations under constant, predictably fluctuating, and randomly fluctuating thermal regimes, we assessed survival and fecundity: a plasticity assay testing flies directly from their evolutionary environments to capture total phenotypic responses, and a common garden assay after two generations of standardized rearing to isolate genetic changes. Strikingly, environmental predictability shaped divergent life-history strategies that were only revealed by comparing our two assays. Populations from predictably fluctuating environments evolved enhanced survival, but this benefit was only visible in the common garden assay, not when tested directly from their evolutionary environment. Conversely, populations from randomly fluctuating environments showed reduced survival in the plasticity assay and consistently lower fecundity in the common garden assay, though this reproductive cost was completely masked in the plasticity assay. These contrasting responses demonstrate that environmental predictability fundamentally determines life-history evolution: predictable variation favors investment in stress-resistant longevity, while unpredictable variation imposes both immediate survival costs and constitutive reproductive constraints. Our findings challenge the traditional view that environmental variation uniformly selects for increased plasticity, instead revealing that the predictability of environmental change determines both the target and mechanism of adaptation. As climate change increases environmental variability and reduces environmental predictability, these insights provide crucial guidance for predicting species persistence and developing effective conservation strategies.

## Introduction

As our planet enters the Anthropocene, species face unprecedented environmental changes that challenge their persistence. Climate change is not only increasing mean temperatures globally but also generating greater thermal anomalies and unpredictability, imposing selective pressures on phenotypes. Species survival depends critically on how they adjust their life-history traits—particularly survival and reproduction—to novel conditions. Phenotypic plasticity has long been recognized as a primary mechanism allowing populations to cope with environmental change, while genetic adaptation was traditionally thought to be too slow for rapid adjustments. However, emerging research reveals that both mechanisms can act in concert, along with transgenerational effects where parental environments influence offspring phenotypes (Diaz et al., 2021; Cavieres et al., 2020).

Life-history traits demonstrate remarkable flexibility in the face of environmental change, responding through both genetic and non-genetic mechanisms (Diaz et al., 2021; Vinton et al., 2022, 2023). The magnitude and nature of these responses vary substantially across species with different life-history strategies (Compagnoni et al., 2021; Tuljapurkar et al., 2009). For instance, *Drosophila* populations show rapid changes in age-specific survival patterns through both plastic (Kubrak et al., 2017) and evolved mechanisms when exposed to temperature variation (Hoffmann et al., 2003; Sørensen et al., 2020). Other studies have revealed profound shifts in reproductive timing that can persist across generations through epigenetic inheritance (DiVito et al., 2023). Despite these insights, disentangling the relative contributions of individual plasticity (Mathur et al., 2017) and evolutionary adaptation across generations (Hoffmann et al., 2023) remains challenging, especially because similar phenotypic outcomes can result from different combinations of these processes (Diamond et al., 2016; Lalejini et al., 2021). Understanding how these adaptive mechanisms interact is crucial for predicting species persistence, yet experimental evidence remains limited (but see Leung et al., 2023; Manenti et al., 2015; Rescan et al., 2022). Traditionally, studies have focused on either plastic or evolutionary responses in isolation, making it difficult to determine their relative importance in adapting to environmental unpredictability (Vinton et al., 2022, 2023).

The role of environmental predictability in shaping these life-history traits adds another layer of complexity to this puzzle (Diaz et al., 2021; Franch-Gras et al., 2018; Vinton et al., 2022, 2023). When environments change in predictable ways—like seasonal temperature cycles—organisms might evolve strong plastic responses that allow precise matching of survival and reproductive strategies to anticipated conditions (Gallegos et al., 2025; Vinton & Vasseur, 2020). What happens when environmental change becomes unpredictable, an increasingly common reality under climate change? Some studies suggest unpredictable variation might select for entirely different life-history strategies (Park et al., 2021), such as bet-hedging mechanisms that sacrifice mean fitness for reduced variance in fitness (Pinceel et al., 2021). However, we lack experimental evidence that clearly distinguishes whether unpredictable environments primarily favor plastic responses that allow individuals to adjust within their lifetime, or evolutionary changes that optimize performance across variable conditions over generations (Vinton et al., 2022). While several recent studies have examined adaptation to environmental predictability (Chevin et al., 2010; Manenti et al., 2016;Sørensen et al., 2020), few have explicitly investigated how predictability affects the relative importance of plastic versus evolutionary adaptation (but see Manenti et al., 2015), especially regarding its differential effects on survival versus reproduction. Since adaptation mechanisms directly influence both the limitations and persistence of adaptive responses, empirically investigating the relationship between environmental predictability and the relative importance of plastic versus evolutionary adaptation is crucial.

**Figure 1.**
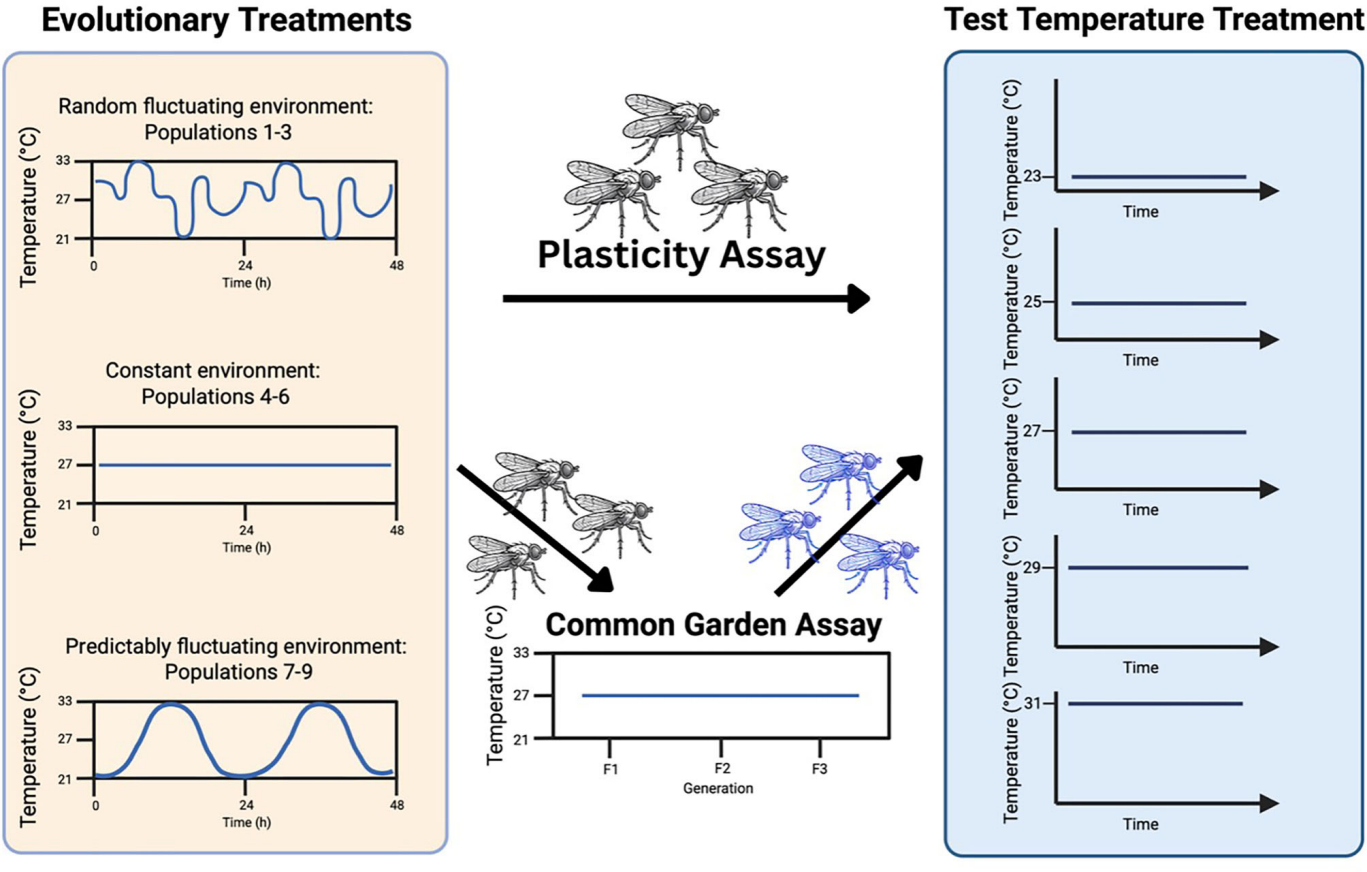
Experimental design for testing fruit fly adaptation to environmental predictability. The left panel reflects thermal regimes experienced by fly populations throughout the experiment: constant (27 °C), predictably fluctuating (27 °C ± 6 °C on a fixed diurnal cycle), and randomly fluctuating (same temperature range but randomly shuffled weekly). The right panel shows the test temperature treatments used in the assays. At each sampling point (parent generations 4, 7, and 10), two types of assays were conducted: (1) a plasticity assay where flies were directly tested across five test temperatures (23, 25, 27, 29, and 31 °C), and (2) a common garden assay where flies were first reared for two generations at 27 °C before testing across the same test temperatures. For each assay condition, survival and fecundity were measured using four replicate vials containing two males and two females per vial. Each thermal treatment included three replicate populations (*n* = 9 populations total).

We address these questions through a novel experimental approach using *Drosophila melanogaster* populations exposed to three distinct thermal environments: constant, predictably fluctuating, and randomly fluctuating temperatures (Figure 1). Our experimental design combines both plasticity (PA) and common garden assays (CG) across multiple generations, enabling us to distinguish between immediate plastic responses and evolutionary changes in survival and reproductive patterns. By measuring age-specific survival and fecundity in populations from each thermal regime, we can systematically evaluate how different patterns of environmental variation influence life-history traits through both plastic and evolutionary mechanisms.

Our experimental design tests two key hypotheses about adaptive responses to thermal predictability, building on previous work showing that predictable environmental variation often favors plastic responses (Oostra et al., 2018; Tufto, 2015).

Hypothesis 1: Environmental variability will drive adaptive evolution, with populations from variable environments evolving different trait values or tolerance breadths compared to constant environments. We predict that populations from variable environments will either (a) evolve different mean performance levels, (b) evolve broader thermal tolerance (maintaining performance across temperatures), or (c) evolve different plastic responses to temperature. Our analyses can distinguish between these outcomes by examining both mean trait differences and reaction norm shapes.

Hypothesis 2: The predictability of environmental variation will determine whether adaptation occurs primarily through plastic responses or genetic evolution. Predictable variation should favor consistent directional selection leading to genetic adaptation, while unpredictable variation should favor mechanisms that maximize flexibility, such as enhanced plasticity or diversifying bet-hedging strategies (e.g., stochastic phenotype switching or increased offspring variability).

Based on these hypotheses, we make the following specific predictions:

- In the PA, we predict that flies from both variable environments will have longer lifespans and higher reproductive outputs across a broader range of temperatures compared to those from constant environments. This response will be strongest in predictably fluctuating populations due to their ability to anticipate environmental changes (Leung et al., 2020; Sánchez-Hernández et al., 2024). It is important to note that this assay captures the combined effects of both evolutionary change and plastic responses, as flies are tested directly from their evolutionary environments without removing parental effects.

- In the CG, where parental and developmental effects are removed, the relationship between environmental predictability and evolutionary change is likely to be more complex. While Hypothesis 2 suggests that predictable environments should show stronger genetic adaptation, theoretical work also suggests that increased plasticity could shield genetic variation from selection, potentially reducing evolutionary responses in these environments compared to randomly fluctuating ones (Ancel, 2000; Bogert, 1949). This creates competing predictions for our CG: in predictable environments, we might observe longer lifespans and higher fecundity across a broader range of temperatures due to consistent selection, or the opposite due to the buffering effects of plasticity. Our approach addresses the practical challenge of distinguishing between multiple adaptive mechanisms that may be differentially favored depending on the pattern of environmental variation (Vinton et al., 2022). By comparing responses between assay types, we can identify which of the predicted outcomes occurred: evolved changes in mean performance (visible in the CG), evolved changes in thermal tolerance breadth, or the importance of environmentally induced responses including parental effects (visible only in the PA). This dual-assay design reveals how environmental predictability shapes both the traits that evolve and the mechanisms through which they are expressed.

## Methods

### Study system and stock

*Drosophila melanogaster* serves as an ideal model organism due to its rapid generation time and extensive array of available genetic tools. We used Dahomey wild-type stock (Wigby & Chapman, 2004), maintained prior to the experiment in large outbred populations with overlapping generations since the 1970s kept at 25 °C with 12:12 hr light:dark cycles in a humidity-controlled room. This base population was kept on standard Lewis medium (Lewis, 1960). To obtain our test populations, eggs were collected from the base population using a standard larval density method (Clancy & Kennington, 2001), and from the eclosed adults, 250 flies were collected to establish each replicate population. We established nine populations total: three replicate populations for each of the three thermal treatments (constant, predictably fluctuating, and randomly fluctuating). These populations were then acclimated to 27 °C for two generations to establish a common thermal baseline (parent generation 0) before initiating the experimental temperature treatments. Each population was maintained in a separate population cage within its designated incubator. All three replicate populations within each treatment experienced identical temperature sequences. For the randomly fluctuating treatment, a single random sequence was generated each Monday and applied to all three replicate populations, ensuring that observed differences were due to environmental predictability rather than different temperature exposures. This design provided three independent replicate populations per thermal treatment, avoiding pseudoreplication. For egg collection and generation transfers, flies from each population cage were temporarily moved to bottles, and for demographic assays, subsamples were placed in vials (see the *Demographic data collection* section).

### Evolutionary environments

Throughout the course of this experiment, fly populations were assigned to one of three temperature regimes (see Figure 1), with all populations maintained under 12:12 hr light:dark cycles. The first was a constant 27 °C regime. The second was a predictably fluctuating regime, on a diurnal fluctuation treatment 27 °C ± 6 °C, which was a stepwise regime with eight steps (21, 24, 27, 30, 33, 30, 27, and 24 °C). Lastly, there was a randomly fluctuating treatment with the same eight values (21, 24, 27, 30, 33, 30, 27, and 24 °C) in the diurnal treatment randomly shuffled using a random choice generator every Monday and the sequence repeated every 24 hr for the week, with each of the eight temperature steps lasting 3 hr.

Each Monday, we generated a new random sequence using R’s sample() function without replacement to randomly order the eight temperature steps (see Supplementary Table 13). This weekly reshuffling ensured no autocorrelation in temperature sequences beyond the 24-hr cycle, preventing flies from adapting to predictable multi-day patterns. The same random sequence was applied to all three replicate populations within the random treatment to ensure that any differences between replicates were not due to different temperature exposures. Over the 11-generation experiment, this resulted in approximately 22 different random temperature sequences, preventing flies from evolving responses to specific temperature patterns beyond the daily cycle. With each temperature step lasting 3 hr and cycling through eight steps daily, flies experienced 112 temperature changes per 14-day generation, ensuring multiple exposures to the full temperature range within each generation. This frequency of temperature change is ecologically relevant for *D. melanogaster*, which experience diurnal temperature fluctuations in nature, and ensures that selection acts across the full range of thermal conditions rather than on single extreme events. The maximum temperature of 33 °C was selected based on its documented role as a stressful but non-lethal temperature for *D. melanogaster*. Flies maintained at 32 °C showed reduced but viable survival and reproduction (Vinton et al., 2025), while 33 °C falls well below the critical thermal maximum (CTmax) of 38–42 °C for this species (Folk et al., 2007; Kellermann et al., 2012). Klepsatel et al. (2016) found no immediate mortality when flies were exposed to 33 °C for 24 hr, though significant metabolic stress was evident through depleted energy reserves. Additionally, Hoffmann et al. (2003) documented that 33 °C induces heat shock protein expression without causing acute mortality during short exposures. Thus, our 3-hr exposure periods at 33 °C provide sufficient thermal stress to generate selection pressure while allowing recovery between temperature cycles.

Fly populations were housed in SciQuip IncuC series cooled incubators. These populations were kept for 11 generations. To maintain non-overlapping generations, 400 eggs per population were collected every 2 weeks from the previous generation and placed in their respective incubator environments to develop. Upon emergence, 250 flies were collected using carbon dioxide anesthesia and transferred to fresh food to replace the previous generation for each respective population. Food was replaced regularly to avoid the development of new larvae. To maintain synchronous generations across all treatments despite temperature-dependent differences in developmental time, we collected eggs from all populations on the same calendar day every 2 weeks. This ensured that all populations experienced the same number of generations (11 total) over the experimental period, though populations in warmer conditions developed from egg to adult faster than those in cooler conditions within each 2-week interval.

### Plasticity assay environments

To assess thermal responses at different points during this experiment, we sampled eggs from populations at four time points: before selection began (parent generation 0, when all populations were at 27 °C) and after 4, 7, and 10 generations of selection in their respective incubator treatments (Figure 1). At each timepoint, we collected eggs from each population and distributed them across five test temperatures with constant-temperature water baths (23, 25, 27, 29, and 31 °C). This PA design captures the combined effects of multiple adaptive mechanisms: within-generation phenotypic plasticity (the individual’s ability to respond to temperature), transgenerational effects including parental effects (where the thermal environment experienced by parents influences offspring phenotypes), and any evolutionary changes that have accumulated over generations. Because flies were tested directly from their evolutionary environments without removing parental effects, the observed responses represent the full suite of adaptive mechanisms available to populations experiencing variable thermal environments. The exact parental temperatures at the time of egg collection varied depending on the position in the temperature cycle for fluctuating treatments, potentially contributing additional variation in offspring phenotypes through parental effects. For each population-by-temperature combination, approximately 200 eggs were placed in one food bottle to develop using a standard larval density method (Clancy & Kennington, 2001). Once these flies emerged as adults, we established four replicate vials per population at each test temperature, with each vial containing two randomly selected males and two females. These flies were maintained at their respective test temperatures in water baths throughout their lifetime.

### Common garden assay environments

To begin the CG, we collected 200 eggs from each of the three populations in each of the three incubator (evolutionary) treatments and transferred them to a low-density food bottle. These were placed in a common garden incubator maintained at 27 °C to develop into adults (F1 generation) using a standard larval density method (Clancy & Kennington, 2001). Fifty males and 50 females were collected using carbon dioxide anesthesia, and after 3 days were moved to a new bottle to lay eggs for a period of 24 hr. F1 adults were removed, and the F2 eggs were allowed to develop and emerge as adults in the population cage. After 3 days, 50 male and 50 female F2 adults were counted using carbon dioxide anesthesia and placed in the cage to lay eggs over a period of 24 hr. This process was repeated to produce the F3 generation eggs, which were subsequently placed into their respective test temperature treatments to develop.

For the test temperature water bath assay, approximately 200 F3 eggs from each population were placed into one food bottle and allowed to develop across five different test temperatures (23, 25, 27, 29, and 31 °C). Once these flies emerged as adults, we established four replicate vials per population at each test temperature, with each vial containing two males and two females. These flies were maintained at their respective test temperatures throughout their lifetime for data collection.

### Demographic data collection

Demographic data were collected from flies in each test temperature treatment. To measure fecundity (average egg production per female), flies were provided with yeast paste and allowed to lay eggs in their vials in their respective test temperatures for 20 hr. After this period, adults were returned to their respective water bath and their eggs were counted under a microscope. To track survival, all adult vials were monitored twice weekly until all four flies in each vial had died.

### Statistical analysis

All statistical analyses were performed in R version 4.4.2. We analyzed survival and fecundity data separately for the PA and CG to examine the distinct effects of immediate and plastic versus evolutionary responses to environmental variation.

We first tested for replicate population effects by including replicate as a factor in preliminary models. Replicate effects were non-significant across all analyses (*p* > 0.05), allowing us to pool data across replicates to increase statistical power. Our analysis tested two primary hypotheses: (1) that environmental variability would affect thermal performance differently than constant conditions, and (2) that the type of variation (predictable vs. random) would produce distinct adaptive responses. We used likelihood ratio tests to compare nested models and evaluate the significance of main effects and interactions. Full model specifications and supporting data visualizations are provided in the Supplementary Material (Supplementary Tables S1–S13 and Supplementary Figures S1–S4).

#### Survival analysis

We fitted Cox proportional hazard models to fly mortality data using the coxph function from the survival package. For both the PA and CG, our initial models included three main predictors: evolutionary environment (predictably fluctuating, randomly fluctuating, or constant), test temperature assay (23, 25, 27, 29, or 31 °C), and parent generation (number of generations spent in the evolutionary environments: 4, 7, or 10). To test for evolved differences in plasticity, we specifically evaluated models including Evolutionary Environment × Test Temperature interactions, which would indicate that populations from different evolutionary environments evolved different reaction norm slopes.

We first tested main effects of these predictors, then evaluated biologically relevant interactions using likelihood ratio tests. Model selection proceeded by comparing nested models using both Akaike Information Criterion (AIC) values and likelihood ratio tests. We considered models with delta AIC values within 1% of the minimum AIC value to be equally well-supported, following the recommendation of using proportional differences rather than absolute differences for model selection (Burnham & Anderson, 2002).

Model adequacy was assessed using concordance statistics and examination of Schoenfeld residuals to verify the proportional hazards assumption.

#### Fecundity analysis

To analyze egg production per female, we used generalized linear models with negative binomial error distributions to account for overdispersion in the count data, which was confirmed by examining the ratio of residual deviance to residual degrees of freedom in initial Poisson models (PA: 12.54, CG: 12.83; ratios > 1 indicate overdispersion). These models were implemented using the glm.nb function from the MASS package. Initial model specification included the same predictors as the survival analysis (evolutionary environment, test temperature, and parent generation), and fly age as an additional predictor to account for changes in fecundity over time.

Models had the functional form:


\begin{eqnarray*}
&&{\mathrm{number \, of \, eggs \, per \, female \sim intercept + \beta 1}}\\
&&\, \times\, {\mathrm{age + \beta 2 \times ag}}{{{\mathrm{e}}}^{\mathrm{2}}} + {\mathrm{error}}
\end{eqnarray*}

to allow for curvilinear relationships of fertility with respect to age. We first tested main effects using likelihood ratio tests, then evaluated interactions based on biological relevance.

For all analyses, we report hazard ratios (HR) with 95% confidence intervals (CI) for survival models, and coefficient estimates with standard errors for fecundity models. *p*-Values less than 0.05 were considered statistically significant, though we detail a range of non-significant effects (*p* < 0.10) when they occur in theoretically meaningful contexts. Full model specifications are provided in the supplementary material.

## Results

Our experiment tracked how fruit fly populations adapted to three different thermal environments over multiple generations: a predictably fluctuating environment, a randomly fluctuating environment, and a constant environment. We assessed adaptation through two different assay types: a PA that tested flies directly from their evolutionary environments, and a CG that removed parental effects by rearing flies for two generations in a standard environment before testing (Figure 1).

### Immediate response to evolutionary environments (plasticity assay)

For survival, likelihood ratio testing showed that adding evolutionary environment significantly improved model fit (χ² = 9.74, df = 2, *p* = 0.008; Supplementary Table 2), with the best model including the interaction between parent generation and test temperature plus independent effects of evolutionary environment (concordance = 0.682, SE = 0.008; Supplementary Table 3). When tested directly from their evolutionary environments, flies showed strong effects of both their immediate thermal experience and their evolutionary history. The evolutionary environment significantly influenced survival, but only for populations from randomly fluctuating conditions, which showed shorter lifespans compared to the reference environment (constant) populations (HR = 1.214, 95% CI: 1.074–1.372, *p* = 0.002). Populations from predictably fluctuating environments showed no significant difference in mortality risk compared to those from constant environments (HR = 1.072, 95% CI: 0.950–1.211, *p* = 0.260).

**Figure 2.**
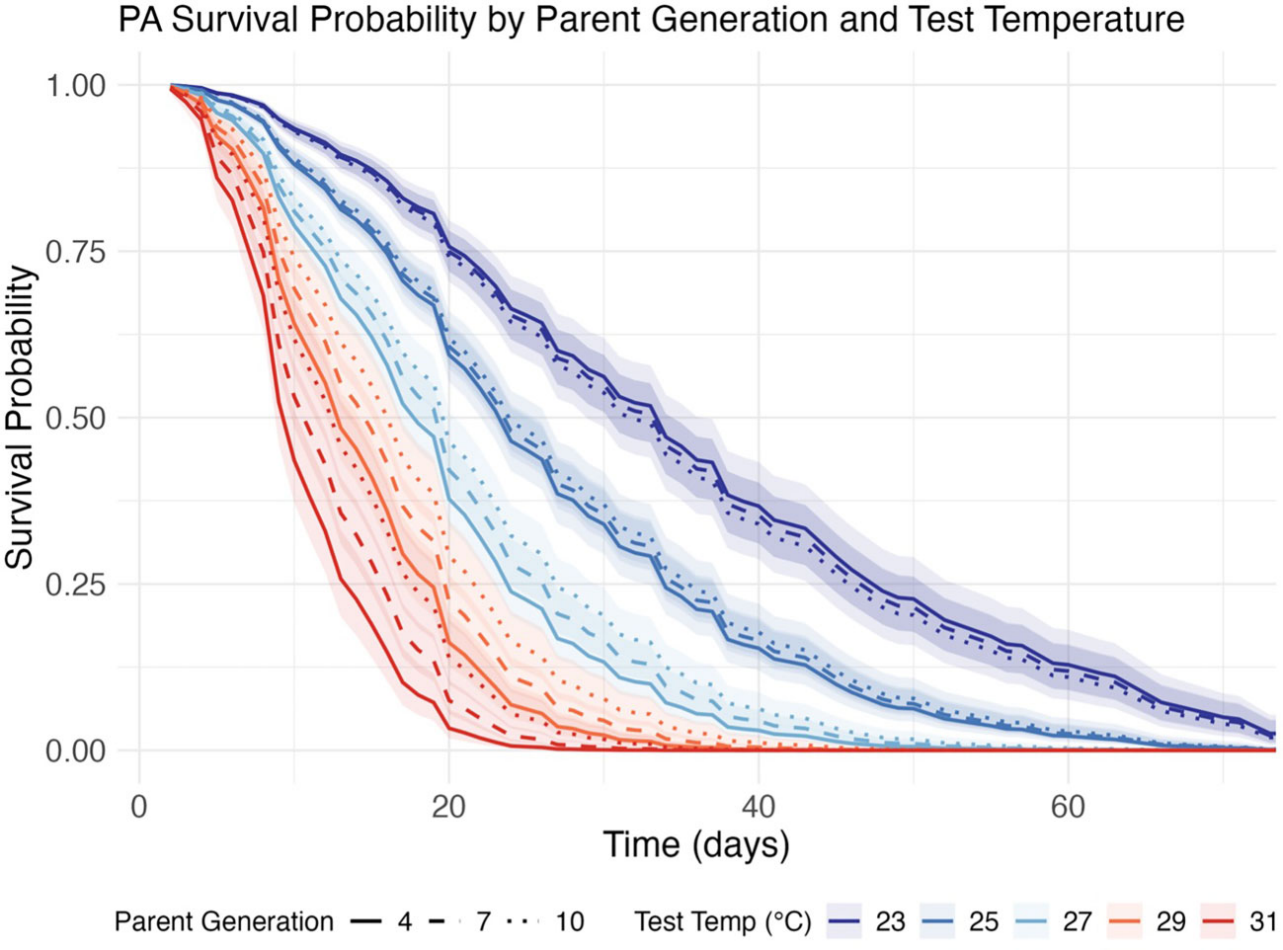
Survival curves from the plasticity assay showing temporal patterns of lifespan in *Drosophila melanogaster* populations tested in the plasticity assay. Lines show the predicted proportion of flies surviving over time at each assay temperature (23, 25, 27, 29, and 31 °C; shown in different colors) for populations sampled after 4, 7, and 10 generations of selection (shown by different line types). Each curve represents pooled data across replicate populations within each treatment combination.

As shown in Figure 2, flies had shorter lifespans with increasing test temperature as expected (HR = 1.440, 95% CI: 1.355–1.531, *p* < 2e–16). The number of parent generations spent in evolutionary environments also decreased mortality risk (HR = 1.362, 95% CI: 1.104–1.680, *p* = 0.004), but this effect was moderated at the lowest test temperature (Parent Generation × Test Temperature interaction HR = 0.987, 95% CI: 0.979–0.995, *p* = 0.001).

**Figure 3.**
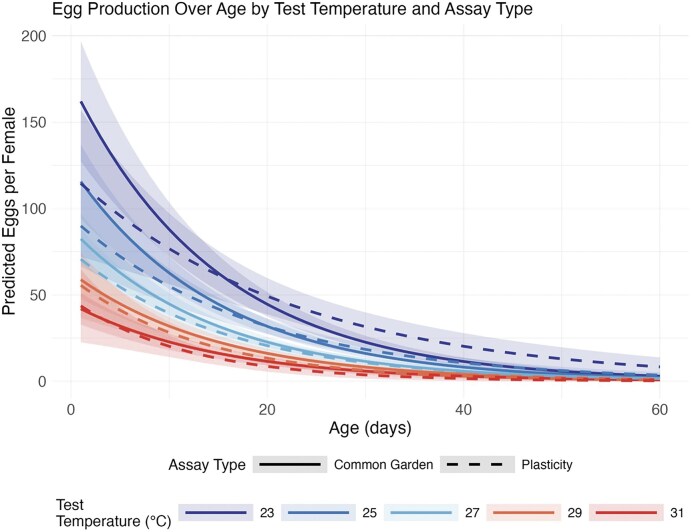
Age-specific egg production patterns in *Drosophila melanogaster* exposed to test temperatures in the plasticity assay. Lines show predicted daily egg counts per female across different ages when tested at five test temperatures (23, 25, 27, 29, and 31 °C), with solid lines representing the common garden assay (CG) and dashed lines representing the plasticity assay (PA). Predictions are derived from the best-fit negative binomial models for each assay type and demonstrate how reproductive output changes with age, with steeper declines observed at higher temperatures in both assays. The Temperature × Age interaction was particularly strong in the common garden assay (coef = −0.0058, *p* = 0.0002), indicating an evolutionary response in age-specific fecundity patterns. Shaded regions represent 95% confidence intervals around model predictions. For the CG, evolutionary environment significantly affected egg production patterns, while in the PA, responses were primarily driven by immediate temperature effects.

We next examined how evolutionary environments affected egg production patterns in the PA. Model selection for fecundity data revealed that the interaction between test temperature and age significantly improved fit (χ² = 10.4, df = 1, *p* = 0.001), with parent generation marginally improving the model (χ² = 2.16, df = 1, *p* = 0.14; Supplementary Tables 8 and 9). The final model included: Age + Parent Generation + Test Temperature + Age × Test Temperature (AIC = 4990.79, θ = 1.21). Egg production in the plasticity assay was primarily influenced by test conditions rather than evolutionary history. The best-fit negative binomial model included test temperature, age, and their interaction, plus parent generations. As shown in Figure 3, egg production as flies aged was negatively impacted by increasing test temperature (Test Temperature × Age interaction: coef = −0.0048, SE = 0.0015, *p* = 0.001). Higher test temperatures also directly reduced egg production (coef = −0.121, SE = 0.027, *p* < 0.0001). The evolutionary environment had no significant effect on immediate egg production patterns, as models including evolutionary environment effects showed poorer fit. Notably, the Evolutionary Environment × Test Temperature interaction was not significant in our model selection (ΔAIC > 19.2 relative to the selected model), indicating that all populations maintained similar plastic responses regardless of their evolutionary history.

### Adaptive response after common garden (common garden assay)

**Figure 4.**
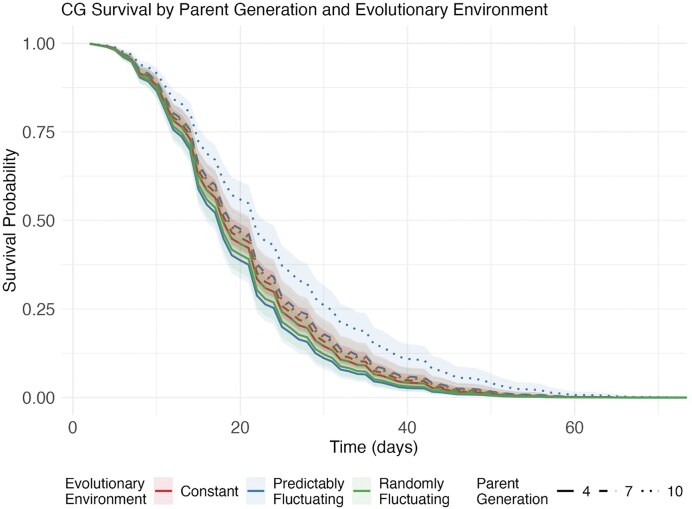
Survival patterns in the common garden assay after two generations of standardized rearing. Lines show the proportion of flies surviving over time from populations evolved in different thermal regimes (constant, predictably fluctuating, and randomly fluctuating; shown by colors) and sampled after different durations of selection (4, 7, and 10 generations; shown by line types). Shaded areas represent 95% confidence intervals around each survival curve. Each curve represents pooled data across replicate populations and water bath temperatures. Survival curves were estimated using a Cox proportional hazards model, revealing a significant interaction between parent generation and evolutionary environment (coef = −0.06586, *p* = 0.0103).

When parental effects were removed through common garden rearing, different patterns emerged. Model selection showed that the interaction between parent generation and evolutionary environment significantly improved fit (χ² = 7.40, df = 2, *p* = 0.025; Supplementary Table 5), yielding the final model: parent Generation + Evolutionary Temperature + Test Temperature + Parent Generation × Evolutionary Temperature (concordance = 0.696, SE = 0.007; Supplementary Table 6). The best-fit survival model showed an interaction between parent generations and evolutionary environment. As visualized in Figure 4, flies from predictably fluctuating environments initially had lifespans shorter than other treatments (HR = 1.480, 95% CI: 1.017–2.155, *p* = 0.041) but evolved to have longer lifespans than the other treatments after 10 generations (Parent Generation × Predictably Fluctuating Environment interaction hazard ratio = 0.936, 95% CI: 0.890–0.985, *p* = 0.010). Evolving in a randomly fluctuating environment had no significant effect on survival probabilities after common garden rearing (HR = 1.143, 95% CI: 0.781–1.673, *p* = 0.493). Test temperature remained a strong predictor of survival (HR = 1.337, 95% CI: 1.306–1.369, *p* < 2e–16).

**Figure 5.**
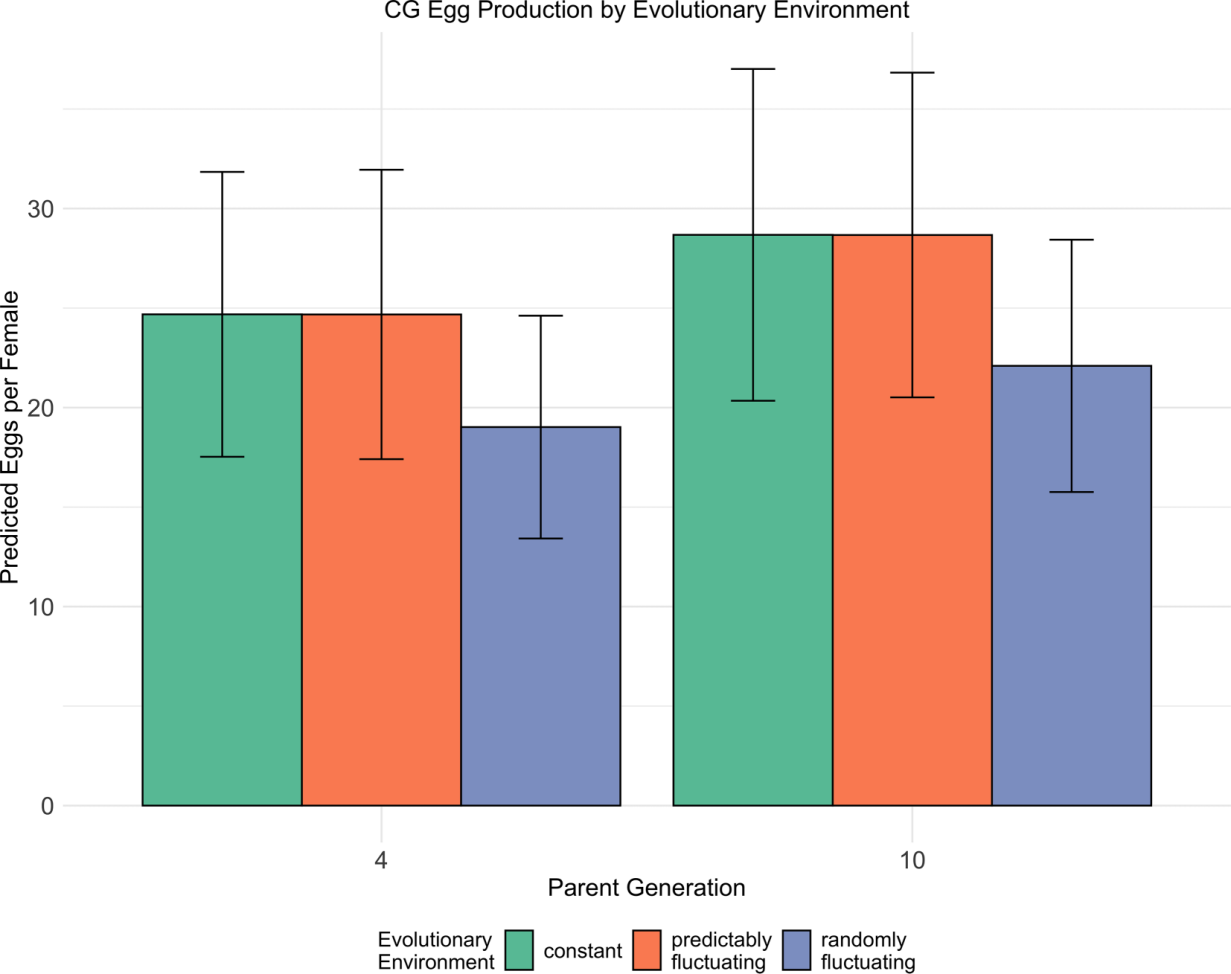
Average predicted egg production per female comparing early (parent generation 4) and late (parent generation 10) generations across different evolutionary environments in the common garden assay. The bars represent mean egg counts, with error bars showing 95% confidence intervals calculated as ± 1.96 standard errors around the mean. Each temperature treatment is shown in a different color, with bars grouped by parent generation to facilitate comparison of evolutionary responses. The y-axis displays the model-predicted average number of eggs laid per female, while the x-axis indicates the parent generation (4 or 10).

Analysis of egg production in the common garden revealed different patterns from survival. For the fecundity models, adding evolutionary environment significantly improved model fit (χ² = 8.95, df = 2, *p* = 0.011), with parent generation showing marginal significance (χ² = 3.42, df = 1, *p* = 0.065; Supplementary Tables 11 and 12). The best model included: Age + Parent Generation + Test Temperature + Evolutionary Environment + Age × Test Temperature (AIC = 4659.33, θ = 1.36). Daily egg counts per female in the CG revealed both environmental and evolutionary effects. As shown in Figure 5, flies showed a marginal increase in fecundity across generations (coef = 0.031, SE = 0.016, *p* = 0.056). Notably, flies from randomly fluctuating environments had significantly lower baseline fecundity (coef = −0.283, SE = 0.095, *p* = 0.003), though they increased at similar rates to other treatments. Flies from randomly fluctuating environments had lower fecundity (coef = −0.283, SE = 0.095, *p* = 0.003) at parent generation 4, while those from predictably fluctuating environments showed no significant difference from constant environmental treatments (coef = −0.020, SE = 0.092, *p* = 0.831). Higher test temperatures reduced egg production (coef = −0.079, SE = 0.028, *p* = 0.005) and accelerated the decline in egg production with age (Test Temperature × Age Interaction: coef = −0.0058, SE = 0.0016, *p* = 0.0002) (Figure 3).

### Environmental context and evolutionary responses

The parallel deployment of PA and CG reveals how environmental context shapes the expression of evolutionary adaptations. The PA integrates all adaptive mechanisms as they function in nature, while the CG isolates constitutive genetic changes.

Temperature universally constrained performance across contexts. Both assays showed remarkably consistent negative effects of high temperature on survival (PA: HR = 1.440; CG: HR = 1.337) and fecundity (PA: β = −0.121; CG: β = −0.079), indicating fundamental thermal limits that transcend evolutionary history.

To test whether populations evolved different plastic responses to temperature, our model selection process evaluated Evolutionary Environment × Test Temperature interactions for both survival and fecundity in each assay (Supplementary Tables 1–12). These interaction terms, which would indicate evolved differences in reaction norm slopes, were not retained in any final models. For example, in the PA survival analysis, models including this interaction had ΔAIC values exceeding our 1% threshold (Supplementary Table 1), and similar patterns occurred for all trait–assay combinations. This indicates that while populations evolved different mean responses to their evolutionary environments, they maintained similar plastic responses to temperature variation—the shape of their thermal reaction norms remained consistent across treatments.

However, the two assays revealed contrasting evolutionary outcomes between predictably fluctuating and randomly fluctuating environments. In the common garden, populations from predictably fluctuating environments evolved enhanced survival at high temperatures (Parent Generation × Predictably Fluctuating Environment interaction: HR = 0.936, *p* = 0.010), a benefit not apparent when tested directly from their evolutionary environment. This suggests that immediate environmental responses may mask underlying genetic adaptation to predictable stress.

All populations showed marginal increases in reproductive output over generations in the common garden (β = 0.031, *p* = 0.056), though populations from randomly fluctuating environments maintained lower baseline fecundity. The absence of Evolutionary Environment × Test Temperature interactions in both assays (all *p* > 0.05) indicates that environmental predictability shaped mean trait values rather than altering thermal reaction norms—populations evolved different elevations but not different slopes in their temperature response curves. Model fit diagnostics for all analyses are provided in Supplementary Tables 3, 6, 9, and 12.

## Discussion

### Environmental predictability determines adaptive strategies

Our findings provide the first clear experimental evidence in *D. melanogaster* that environmental predictability fundamentally determines which life-history traits evolve. While Leung et al. (2023) demonstrated reduced plasticity across molecular and morphological levels in *Dunaliella salina* under unpredictable conditions, and Manenti et al. (2015)found unchanged plasticity levels in *D. simulans*, our results reveal a more nuanced pattern: predictability shapes not just the magnitude but the target of adaptation. Specifically, predictable environments favored the evolution of enhanced survival at high temperatures, while unpredictable environments favored altered reproductive strategies—a life-history dichotomy not previously demonstrated experimentally. The contrasting responses in survival and fecundity between predictably and randomly fluctuating environments suggest that the predictability of environmental variation plays a crucial role in shaping adaptive strategies (Bogaerts‐Márquez et al., 2021; Gallegos et al., 2025; Machado et al., 2021 ). This aligns with theoretical predictions about how environmental predictability influences the evolution of plastic responses and genetic adaptation (Leung, et al., 2020).

### Life-history trade-offs under thermal stress

In terms of survival, flies from the predictably fluctuating environments evolved longer lifespans at high temperatures, but only when tested after common garden rearing. This suggests that genetic adaptation occurred in response to predictable environmental variation, though these benefits were masked by immediate environmental effects in the PA. This pattern aligns with previous work showing that predictable environments can favor the evolution of specific adaptive mechanisms (Feng et al., 2024; Lind et al., 2020; Zhou et al., 2024), but also raises questions about why these benefits were not consistent across assay types. This contrasts with Rescan et al. (2022), who found that *D. salina* from predictable environments showed stronger acclimation effects (larger *k* values) but not necessarily improved survival. Our finding that these survival benefits were masked in the PA highlights the importance of our dual-assay approach—previous single-assay studies (e.g., Manenti et al., 2015) may have missed such cryptic evolutionary responses. One possibility is that parental effects or other forms of transgenerational plasticity may actually counteract some evolved adaptations, perhaps as a bet-hedging strategy in variable environments (Moore et al., 2019; Plaistow & Benton, 2009; Räsänen & Kruuk, 2007).

The impact on fecundity reveals a contrasting pattern between evolutionary environments. While populations from predictably fluctuating environments showed no change in reproductive output, those from randomly fluctuating environments evolved constitutively lower fecundity in the CG (coef = −0.283, *p* = 0.003). However, this genetic cost was completely masked by parental effects in the PA, suggesting a complex interaction between different forms of adaptation. These findings contribute to our understanding of how organisms optimize their reproductive strategies in variable environments (Schaum et al., 2022; Winemiller & Rose, 1992), particularly regarding the balance between immediate and transgenerational responses. The fact that only random, not predictable, variation affected fecundity challenges existing theories about adaptive responses to environmental predictability (Oostra et al., 2018; Sánchez-Hernández et al., 2024). This reproductive cost under unpredictable conditions may reflect trade-offs in resource allocation: (1) maintaining broad stress tolerance systems may divert energy from reproduction (Sørensen et al., 2003); (2) random thermal variation might prevent reproductive optimization (Folguera & Bozinovic, 2009; Walsh et al. 2021); or (3) unpredictable environments might favor conservative bet-hedging strategies that reduce per-capita reproductive investment (Crean & Marshall, 2009). The physiological basis may involve changes in cellular stress responses and resource allocation patterns that prioritize stress resistance over reproductive output when facing unpredictable thermal stress.

Our results show that variable environments impact different life-history traits depending on both the predictability of environmental variation and the timeframe of adaptive responses. While predictable variation appeared to favor enhanced survival rates, random variation influenced reproductive patterns, suggesting that different forms of environmental variation may select for distinct life-history strategies (Dey et al., 2016; Giaimo et al., 2019; Griskevicius et al., 2011). These differential responses—survival benefits under predictable variation versus reproductive costs under random variation—may reflect principles of demographic buffering, where populations minimize fitness impacts by reducing sensitivity in the vital rates most strongly influencing population growth (Boyce et al., 2006; Pfister, 1998). In our experiment, different thermal regimes appear to trigger different vital rates being affected—enhanced survival under predictable variation and reduced fecundity under random fluctuations. This trade-off may represent a fundamental constraint on adaptation to variable environments, with implications for understanding how species might respond to increasing environmental variability under climate change.

### Distinguishing plastic from evolutionary responses

The contrast between our PA and CG reveals important insights about the relative roles of parental effects and evolutionary change in adaptation to variable environments. The fact that some adaptive benefits were only visible after removing parental effects suggests that transgenerational plasticity may sometimes mask or counteract evolutionary adaptations (Vijendravarma et al., 2013; Vinton et al., 2022). This finding has important implications for how we study and interpret adaptive responses to environmental change, as immediate plastic responses may not always align with longer-term evolutionary trajectories (Kelly et al., 2019; Mlambo et al., 2024; Sturiale et al., 2023). The absence of an Evolutionary Environment × Test Temperature interaction in both assays indicates that populations evolved different mean responses but maintained similar plastic responses to temperature variation. Thus, the CG reveals evolutionary changes in trait means after removing parental effects, rather than evolved differences in plasticity per se. While theory suggests that predictable environments could favor the evolution of enhanced plasticity (Leung et al., 2020; Tufto, 2015), our results indicate that environmental predictability instead influenced which traits evolved (survival vs. fecundity) rather than the degree of plasticity in those traits.

Our findings may also reflect how different patterns of environmental variation affect the maintenance of genetic diversity. While we did not directly measure genetic variation, both theory and experimental work suggest that fluctuating environments typically maintain more genetic variation than constant ones (Abdul-Rahman et al., 2021; Bürger et al., 2002). The effect of environmental predictability on genetic diversity remains complex: while predictable fluctuations might allow populations to track optimal genotypes over time, random fluctuations could either maintain diversity through inconsistent selection or favor generalist genotypes that perform adequately across all conditions (Vinton & Vasseur, 2020). This creates an apparent paradox in our results: if randomly fluctuating environments preserve more genetic variation, why did these populations show reduced performance compared to those from predictably fluctuating environments? One explanation may lie in the nature of selection under different types of environmental predictability. In predictably fluctuating environments, consistent selective pressures could drive the accumulation of beneficial genetic variants while still maintaining enough variation to cope with regular temperature changes. In contrast, random fluctuations might preserve a broader range of genetic variants but prevent the establishment of consistently advantageous combinations. This interpretation aligns with previous work showing that highly autocorrelated (predictable) environments can increase genetic differentiation (Ranta et al., 2008), potentially accelerating local adaptation. These patterns highlight the importance of considering how environmental predictability shapes microevolution through standing genetic variation, rather than focusing solely on new mutations, especially over relatively short experimental timeframes. Future studies testing different types of random variation, including sequences with varying levels of autocorrelation, would help determine the generality of evolutionary responses to environmental unpredictability.

Our use of a long-maintained laboratory population warrants consideration when interpreting these results. The Dahomey stock’s extended maintenance at constant conditions (25 °C for 20+ years) may have influenced both the standing genetic variation available for selection and the baseline plasticity levels, potentially affecting the evolutionary responses we observed. While this provides a controlled starting point for examining evolutionary responses, future studies using recently collected wild populations or multiple independent stocks would help validate the generality of our findings regarding environmental predictability and adaptive evolution. Additionally, our cross-generation comparisons lacked internal reference controls tested at each timepoint, making it difficult to fully separate evolutionary changes from potential temporal variation in laboratory conditions, though the treatment-specific patterns we observed (particularly the divergent responses in survival vs. fecundity) suggest genuine evolutionary responses rather than simple batch effects. Additionally, while our three replicate populations per treatment showed consistent responses, more extensive replication would enable nested analyses and better resolution of population-level variation. Future work could also explore how the interaction between circadian rhythms and temperature cycles influences evolutionary responses, as thermal stress experienced during active versus inactive periods may generate different selective pressures—an interesting layer of complexity that could shape adaptation to variable environments.

A striking, but not entirely unsurprising aspect of our results was the consistent negative effect of high temperatures across all conditions, regardless of evolutionary history or assay type. This suggests fundamental constraints on thermal adaptation (Miquel et al., 1976; Mitchell & Hoffmann, 2010; but see Gilchrist & Huey, 2001), which may limit organisms' ability to cope with extreme temperatures even when they regularly experience thermal variation. These constraints have important implications for understanding species' responses to climate change, particularly as both average temperatures and temperature variability increase (Taff et al., 2023). The consistency of these temperature effects across our experiments suggests that immediate physiological limitations outweigh both plastic and evolved adaptations.

### Implications for climate change adaptation

These results suggest that fruit flies may adapt to thermal variability through both plastic and evolved responses, with the pattern of environmental variation potentially shaping which adaptive mechanisms predominate. More specifically our dual-assay approach revealed that populations adapted to predictable fluctuations evolved enhanced heat-stress resistance that was only detectable after removing parental effects, while those from unpredictable environments evolved altered reproductive strategies visible only in the PA. This methodological insight—that evolutionary adaptations can be masked by opposing plastic responses—suggests that single-assay assessments may dramatically underestimate adaptive potential or misidentify which traits are evolving. The removal of parental effects through common garden rearing reveals different patterns of response to predictable versus random environmental variation, though the relative contributions of evolutionary adaptation versus other transgenerational effects remain to be fully resolved. These findings have important implications for conservation biology and predictions about species persistence under climate change. The different adaptive responses to predictable versus random variation suggest that increasing environmental unpredictability may pose unique challenges for species persistence. Conservation strategies might need to consider not just the magnitude of environmental change, but also its predictability. Additionally, our results suggest that species' adaptive potential may depend on complex interactions between immediate plastic responses, transgenerational effects, and evolutionary change, making it crucial to consider multiple timeframes when assessing vulnerability to environmental change.

## Supplementary Material

qraf052_Supplemental_File

## Data Availability

The data and code that support the findings of this study are available in Dryad at https://doi.org/10.5061/dryad.2jm63xt3v
