# Supplementary material for: Environmental predictability drives different routes to adaptation
Source: Evol Lett. 2025 Dec 31;10(2):173–85. doi: 10.1093/evlett/qraf052 (PMC13043901; doi:10.1093/evlett/qraf052)

Supplementary Material

Supplementary Table 1: Plasticity Assay Survival optimal models.

Supplementary Table 1 summarizes the candidate Cox proportional hazards models considered for the plasticity assay survival analysis. For each model, the table lists the Akaike Information Criterion (AIC), the difference in AIC (ΔAIC) relative to the best model, the log-likelihood, and the number of estimated parameters. Note that models including Evolutionary Environment × Test Temp interactions (rows X-Y) consistently showed poor fit relative to simpler models, indicating no evolution of differential plasticity between treatments.

The optimal model is defined as the one with the lowest AIC value (here, 19202.16), which in this case is the model including the Parent Gen * Test Temp interaction plus an independent effect of Evolutionary Environment. Models with ΔAIC values within 1% of the minimum AIC were considered to have equivalent support. We further refined our model selection by comparing nested models using likelihood ratio tests (see Supplementary Table 2), ensuring that we chose the most parsimonious model that did not significantly differ from more complex alternatives.

Model	AIC	Delta_AIC	LogLik	n_params
Parent Gen * Test Temp + Evolutionary Environment	19202.16	0.000000	-9596.081	5
Parent Gen * Test Temp	19207.91	5.743940	-9600.953	3
Parent Gen + Evolutionary Environment + Test Temp	19210.46	8.296882	-9601.229	4
Parent Gen * Evolutionary Environment * Test Temp	19210.82	8.657224	-9594.410	11
Evolutionary Environment * Test Temp + Parent Gen	19212.73	10.572866	-9600.367	6
Parent Gen * Evolutionary Environment + Test Temp	19214.44	12.282517	-9601.222	6
Parent Gen + Test Temp	19216.09	13.925461	-9606.044	2
Test Temp + Evolutionary Environment	19218.32	16.157535	-9606.160	3

Supplementary Table 2: Analysis of Deviance for Nested Cox Models in the Plasticity Assay.

This table compares two nested Cox proportional hazards models fitted to the plasticity assay survival data.

- Model 1 includes the interaction between parent generation and test temperature (i.e., ~ *Parent Gen * Test Temp*).
- Model 2 extends Model 1 by adding the main effect of incubator temperature (i.e., ~ *Parent Gen + Test Temp + Evolutionary Environment + Parent Gen:Test Temp*).

The analysis of deviance shows that Model 2 has a higher log-likelihood (−9596.1) compared to Model 1

(−9601.0). The likelihood ratio test yields a chi-squared statistic of 9.7439 on 2 degrees of freedom (p = 0.007658), indicating that the inclusion of the incubator temperature term significantly improves the model fit.

Signif. codes: 0 '' 0.001 '*' 0.01 '*' 0.05 '.' 0.1 ' ' 1*

Model	loglik	Chisq	Df	Pr(>|Chi|)
1	-9601.0			
2	-9596.1	9.7439	2	0.007658 **

Supplementary Table 3: Summary of the Optimal Cox Proportional Hazards Model for the Plasticity Assay.

This table presents the full output of the optimal Cox model selected for the plasticity assay (n = 1556, with 1556 events). The model includes the following predictors:

- Parent Gen (number of generations in the evolutionary environment),
- Test Temp (test temperature in °C),
- Evolutionary Environment (categorical variable with levels "Predictable" and "Random"; the constant environment serves as the reference), and
- the interaction between Parent Gen and Test Temp.

For each predictor, the table shows the estimated coefficient, the exponentiated coefficient (hazard ratio), standard error, z-value, and the corresponding *p*-value. For example, a one-unit increase in test temperature is associated with a 44.0% increase in mortality risk (hazard ratio = 1.440, 95% CI: 1.3547–1.531, p < 2e-16). In addition, the table includes diagnostic statistics:

- Concordance = 0.682 (SE = 0.008),
- Likelihood ratio test = 577.8 on 5 df, p < 2e-16,
- Wald test = 571.7 on 5 df, p < 2e-16, and
- Score (logrank) test = 618.8 on 5 df, p < 2e-16.

These diagnostics confirm the model’s overall fit and its predictive power. Full model selection details are provided in Supplementary Tables 1 and 2.

	coef	exp(coef)	se(coef)	z	Pr(>|z|)	exp(coef)	exp(-coef)	lower .95	upper .95
Parent Gen	0.308713	1.361672	0.107071	2.883	0.00394 **	1.3617	0.7344	1.1039	1.680
Test Temp	0.364629	1.439979	0.031147	11.707	< 2e-16 ***	1.4400	0.6945	1.3547	1.531
Evolutionary Environment Predictable	0.069759	1.072250	0.061953	1.126	0.26017	1.0722	0.9326	0.9496	1.211
Evolutionary Environment Random	0.193962	1.214050	0.062522	3.102	0.00192 **	1.2140	0.8237	1.0740	1.372
Parent Gen:Test Temp	0.012894	0.987189	0.004019	-3.208	0.00134 **	0.9872	1.0130	0.9794	0.995

Supplementary Table 4: Common Garden Survival Optimal Model.

This table summarizes the candidate Cox proportional hazards models considered for the common garden assay survival data. For each model, the table reports the Akaike Information Criterion (AIC), the difference in AIC

(ΔAIC) relative to the best model, the log-likelihood, and the number of estimated parameters. Models were built using different combinations of the three predictors:

- Parent Gen (number of generations in the evolutionary environment),
- Evolutionary Environment (categorical variable with levels representing the evolutionary thermal treatments), and
- Test Temp (the test temperature in °C).

Models with ΔAIC values within 1% of the minimum AIC were considered to have equivalent support. In this analysis, the best model (lowest AIC = 18886.13) was the one including the interaction between Parent Gen and Evolutionary Environment, plus the main effect of Test Temp (i.e., *Parent Gen * Evolutionary Environment + Test Temp*), which required 6 parameters and yielded a log-likelihood of –9437.063. The table lists several alternative models along with their ΔAIC values, illustrating the relative fit of each candidate. This information, together with subsequent likelihood ratio tests (see Supplementary Table 5), guided the selection of the most parsimonious model that best explains survival in the common garden assay.

Model	AIC	Delta_AIC	LogLik	n_params
Parent Gen * Evolutionary Environment + Test Temp	18886.13	0.000000	-9437.063	6
Parent Gen + Evolutionary Environment + Test Temp	18889.52	3.396489	-9440.761	4
Parent Gen + Test Temp	18889.56	3.434534	-9442.780	2
Parent Gen * Test Temp	18891.42	5.294663	-9442.710	3
Parent Gen * Test Temp + Evolutionary Environment	18891.45	5.327425	-9440.726	5
Evolutionary Environment * Test Temp + Parent Gen	18893.08	6.949964	-9440.538	6
Parent Gen * Evolutionary Environment * Test Temp	18894.47	8.344026	-9436.235	11

Supplementary Table 5: Analysis of Deviance for Common Garden Survival Models

This table compares two nested Cox proportional hazards models for the common garden assay, where the response is the survival object (surv_obj_CG).

- **Model 1** includes the main effects of Parent Gen, Evolutionary Environment, and Test Temp (i.e., ~ *Parent Gen + Evolutionary Environment + Test Temp*).
- **Model 2** adds the interaction between Parent Gen and Evolutionary Environment (i.e., ~ *Parent Gen + Evolutionary Environment + Test Temp + Parent Gen:Evolutionary Environment*).

Model 1 has a log-likelihood of –9440.8, while Model 2 shows an improved log-likelihood of –9437.1. The

likelihood ratio test comparing these two models yields a chi-squared statistic of 7.3965 with 2 degrees of freedom (p = 0.02477), indicating that the inclusion of the Parent Gen:Evolutionary Environment interaction significantly improves model fit.

Signif. codes: 0 “’ **0.001 ‘’** 0.01 *‘’ 0.05 ‘.’ 0.1 ‘ ’ 1*

Model	loglik	Chisq	Df	Pr(>|Chi|)
1	-9440.8			
2	-9437.1	7.3965	2	0.02477 *

Supplementary Table 6: Summary of the Optimal Cox Model for Common Garden Survival Data

This table presents the full output of the optimal Cox proportional hazards model fitted to the common garden survival data (n = 1537, with 1537 events). The final model includes the following predictors:

- Parent Gen: Number of generations spent in the evolutionary environment.
- Test Temp: Test temperature (°C).
- Evolutionary Environment Predictable and Evolutionary Environment Random: Indicator variables for the Predictable and Random incubator treatments (with the constant environment as the reference).
- Parent Gen:Evolutionary Environment Predictable and Parent Gen:Evolutionary Environment Random: Interaction terms between Parent Gen and the incubator treatment indicators.

For each predictor, the table reports the estimated coefficient, its exponentiation (hazard ratio), standard error, z-value, and the corresponding *p*-value. For example:

- Test Temp: A coefficient of 0.29030 (hazard ratio = 1.33683, 95% CI: 1.3057–1.3687, $p < 2e-16$ ***) indicates that each 1°C increase in test temperature is associated with a 33.7% increase in mortality risk.
- Evolutionary Environment Predictable: With a coefficient of 0.39227 (hazard ratio = 1.48034, 95% CI: 1.0169–2.1549, $p = 0.0406$ *), this term signifies a significant increase in mortality risk relative to the

constant environment.

- Parent Gen:Evolutionary Environment Predictable: A coefficient of –0.06586 (hazard ratio = 0.93626, 95% CI: 0.8903–0.9846, *p*= 0.0103 *) suggests that the effect of additional generations is slightly moderated under the Predictable treatment.

Additional model diagnostics are as follows:

- Signif. codes: 0 ‘’ *0.001 ‘’ 0.01* ‘’ 0.05 ‘.’ 0.1 ‘ ’ 1
- Concordance = 0.696 (se = 0.007)
- Likelihood ratio test = 616.8 on 6 df, p < 2e-16
- Wald test = 595.3 on 6 df, p < 2e-16
- Score (logrank) test = 644 on 6 df, p < 2e-16

These diagnostics confirm the overall robustness and predictive power of the model. Full details of model selection and further diagnostics are provided in Supplementary Tables 4 and 5.

	coef	exp(coef)	se(coef)	z	Pr(>|z|)	exp(coef)	exp(-coef)	lower .95	upper .95
Parent Gen	-0.01574	0.98438	0.01816	-0.867	0.3861	0.9844	1.0159	0.9500	1.0201
Evolutionary Environment Predictable	0.39227	1.48034	0.19158	2.048	0.0406 *	1.4803	0.6755	1.0169	2.1549

Evolutionary Environment Random	0.13334	1.14264	0.19447	0.686	0.4929	1.1426	0.8752	0.7805	1.6728
Test Temp	0.29030	1.33683	0.01203	24.126	<2e-16 ***	1.3368	0.7480	1.3057	1.3687
Parent Gen:Evolutionary Environment Predictable	-0.06586	0.93626	0.02568	-2.565	0.0103 *	0.9363	1.0681	0.8903	0.9846
Parent Gen:Evolutionary Environment Random	-0.01210	0.98798	0.02606	-0.464	0.6424	0.9880	1.0122	0.9388	1.0397

Supplementary Table 7: Plasticity Assay Egg/Female Optimal Model

This table summarizes the candidate negative binomial generalized linear models used to explain egg

production per female in the plasticity assay. For each model, the Akaike Information Criterion (AIC), the difference in AIC (ΔAIC) relative to the best model, the log-likelihood, and the number of estimated parameters (n_params) are reported.

Candidate models were built using different combinations of the predictors – Test Temp, Age, Parent Gen, and Evolutionary Environment – including main effects and interactions. Models with ΔAIC values within 1% of the minimum AIC were considered to have similar support. In this analysis, the optimal model was identified as:

> Test Temp * Age + Parent Gen (AIC = 4990.789, ΔAIC = 0.0000000, LogLik = –2489.395, n_params = 5)

This model incorporates the interaction between test temperature and age, with an additive effect of parent generation. Alternative candidate models (e.g., those that exclude Parent Gen or include additional terms like Evolutionary Environment) are presented for comparison. The modest ΔAIC value (0.1589) for the model with only the Test Temp * Age interaction indicates that while adding Parent Gen slightly improves the model fit, other combinations (such as including Evolutionary Environment) do not result in a better balance of fit and complexity.

Detailed parameter estimates and tests of significance for the optimal model are provided in Supplementary Table 8.

Model	AIC	Delta_AIC	LogLik	n_params
Test Temp * Age + Parent Gen	4990.789	0.0000000	-2489.395	5
Test Temp * Age	4990.948	0.1588725	-2490.474	4
Test Temp * Age + Parent Gen + Evolutionary Environment	4994.462	3.6724818	-2489.231	7
Test Temp * Age + Evolutionary Environment	4994.627	3.8377664	-2490.313	6
Parent Gen * Test Temp * Age	4995.097	4.3075970	-2488.548	8
Age + Test Temp	4997.662	6.8731589	-2494.831	3
Parent Gen + Test Temp + Age	4997.704	6.9148794	-2493.852	4
Test Temp + Parent Gen + Age	4997.704	6.9148794	-2493.852	4
Parent Gen * Test Temp + Age	4998.499	7.7098138	-2493.249	5
Evolutionary Environment * Test Temp * Age + Parent Gen	5001.447	10.6574740	-2486.723	13
Evolutionary Environment + Test Temp + Age	5001.603	10.8144023	-2494.802	5

Supplementary Table 8: Likelihood Ratio Test of Negative Binomial Models

Response: Egg_Count_Per_Female

LR stat. = 2.158872, Pr(Chi) = 0.1417487

This table compares two negative binomial models fitted to the egg production data (Egg_Count_Per_Female) from the plasticity assay.

- Model 1: Includes the interaction between Test Temp and Age (i.e., *Test Temp * Age*). It has a dispersion parameter (theta) of 1.200584, a log-likelihood of –4980.948, and 572 residual degrees of freedom.
- Model 2: Extends Model 1 by adding Parent Gen as an additional predictor (while retaining the Test Temp:Age interaction). This model has a theta of 1.206964, a log-likelihood of –4978.789, and 571 residual degrees of freedom.

The likelihood ratio test comparing Model 1 and Model 2 yields an LR statistic of 2.158872 on 1 degree of freedom, with a corresponding *p*-value of 0.1417487. This indicates that adding Parent Gen does not significantly improve the model fit ($p > 0.05$).

Model	theta	Resid. df	2 x log-lik	Test	df
Test Temp * Age	1.200584	572	-4980.948		
Test Temp + Age + Parent Gen + Test Temp:Age	1.206964	571	-4978.789	1 vs 2	1

Supplementary Table 9: Summary of the Best Negative Binomial Model (Plasticity Assay)

This table reports the final negative binomial model fitted to egg production per female (data_PA) using a log

link. The model includes Test Temp, Age, Parent Gen, and their interaction (Test Temp × Age). Key findings include a highly significant negative effect of Test Temp (p = 9.26e-06) and a significant interaction between Test Temp and Age (p = 0.00119), while the effect of Age is marginally significant (p = 0.08982) and Parent Gen is not significant (p = 0.15177).

Additional model diagnostics are as follows:

Signif. codes: 0 ‘’ *0.001 ‘’ 0.01* ‘’ 0.05 ‘.’ 0.1 ‘ ’ 1

Dispersion parameter: 1.207 (SE = 0.0839)

Null deviance: 1013.87 (575 df)

Residual deviance: 726.53 (571 df)

AIC: 4990.8

2 x log-likelihood: –4978.7890

Number of Fisher Scoring iterations: 1

	Estimate Std.	Error	z value	Pr(>|z|)
(Intercept)	7.437697	0.726060	10.244	< 2e-16 ***
Test Temp	-0.121244	0.027345	-4.434	9.26e-06 ***
Age	0.062636	0.036924	1.696	0.08982 .
Parent Gen	0.023591	0.016459	1.433	0.15177
Test Temp:Age	-0.004770	0.001472	-3.240	0.00119 **

Supplementary Table 10: Common Garden Egg/Female Optimal Model Comparison

This table presents candidate negative binomial regression models predicting egg production per female in the common garden assay. Models were compared using the Akaike Information Criterion (AIC), with those within a meaningful AIC difference (ΔAIC) considered equally supported. The table lists, for each candidate model, the AIC, ΔAIC relative to the best model, log-likelihood, and number of estimated parameters.

The optimal model is:

Test Temp * Age + Parent Gen + Evolutionary Environment

AIC = 4659.333, ΔAIC = 0.000000, LogLik = –2321.666, n_params = 7

Other candidate models—with varying combinations of predictors and interaction terms—yielded higher AIC values. For example, the model Test Temp * Age + Evolutionary Environment has a ΔAIC of 1.415552 (n_params = 6), and more complex models (e.g., including three-way interactions) resulted in even higher ΔAIC values and greater model complexity. These comparisons demonstrate that the optimal model achieves the best balance between goodness-of-fit and parsimony for the common garden egg production data.

Model	AIC	Delta_AIC	LogLik	n_params
Test Temp * Age + Parent Gen + Evolutionary Environment	4659.333	0.000000	-2321.666	7
Test Temp * Age + Evolutionary Environment	4660.748	1.415552	-2323.374	6
Evolutionary Environment * Test Temp * Age + Parent Gen	4662.390	3.057761	-2317.195	13
Evolutionary Environment * Test Temp * Age	4664.346	5.013435	-2319.173	12
Parent Gen * Test Temp * Age + Evolutionary Environment	4664.931	5.597930	-2321.465	10
Test Temp * Age + Parent Gen	4665.690	6.357185	-2326.845	5
Evolutionary Environment * Age + Parent	4665.729	6.396712	-2323.865	8

Gen + Test Temp				
Test Temp * Age	4666.297	6.964099	-2328.148	4
Parent Gen * Evolutionary Environment * Age + Test Temp	4666.562	7.228912	-2319.281	13
Evolutionary Environment * Age + Test Temp	4666.593	7.260111	-2325.296	7
Parent Gen + Evolutionary Environment + Test Temp + Age	4668.473	9.140475	-2327.237	6

Supplementary Table 11: Likelihood Ratio Test of Negative Binomial Models

Response: Egg_Count_Per_Female

This table compares two nested negative binomial models for predicting egg production per female.

- **Model 1:** Includes Test Temp, Age, Evolutionary Environment, and the Test Temp × Age interaction.
- **Model 2:** Extends Model 1 by also including Parent Gen.

Model 1 has a theta of 1.344211, 532 residual degrees of freedom, and a 2× log-likelihood of –4646.748, while Model 2 shows a theta of 1.355248, 531 residual degrees of freedom, and a 2× log-likelihood of –4643.333. The likelihood ratio test comparing these models yields an LR statistic of 3.415552 on 1 degree of freedom, with a corresponding *p*-value of 0.06458482. This result indicates that adding Parent Gen leads to a marginal improvement in model fit; however, the improvement does not reach conventional significance at the 0.05 level.

Model	theta	Resid. df	2 x log-lik	Test	df

Test Temp + Age + Evolutionary Environment + Test Temp:Age	1.344211	532	-4646.748		
Test Temp + Age + Parent Gen + Evolutionary Environment + Test Temp:Age	1.355248	531	-4643.333	1 vs 2	1

Supplementary Table 12: Summary of the Best Negative Binomial Model (Common Garden Egg Production)

This table displays the final model fitted to the common garden egg production data (data_CG) using a negative binomial regression with a log link. The model includes the predictors Test Temp, Age, Parent Gen, Evolutionary Environment (with “Predictable” and “Random” indicating treatments relative to the constant environment), and the interaction between Test Temp and Age.

Additional model diagnostics are:

Signif. codes: 0 ‘’ *0.001 ‘’ 0.01* ‘’ 0.05 ‘.’ 0.1 ‘ ’ 1

Dispersion parameter (Negative Binomial(1.3552) family): 1

Null deviance: 1013.87 on 575 degrees of freedom

Residual deviance: 726.53 on 571 degrees of freedom

AIC: 4990.8

Number of Fisher Scoring iterations: 1

Theta: 1.3552 (SE = 0.0973)

2 x log-likelihood: –4643.3330

These diagnostics confirm that the model adequately fits the data while accounting for overdispersion in egg production counts.

	Estimate	Std. Error	z value	Pr(>|z|)
(Intercept)	6.548385	0.744033	8.801	< 2e-16 ***
Test Temp	-0.078576	0.028205	-2.786	0.005338 **
Age	0.077461	0.039395	1.966	0.049267 *
Parent Gen	0.030843	0.016151	1.910	0.056180 .
Evolutionary Environment Predictable	-0.019720	0.092354	-0.214	0.830920
Evolutionary Environment Random	-0.283065	0.094992	-2.980	0.002883 **
Test Temp:Age	-0.005835	0.001578	-3.698	0.000217 ***

Model Performance Metrics

Model fit was assessed using concordance statistics for Cox proportional hazards models and dispersion parameters (theta) for negative binomial models. For survival analyses, the plasticity assay models showed concordance of 0.682 (SE = 0.008), while common garden models showed concordance of 0.696 (SE = 0.007). For fecundity analyses, dispersion parameters were 1.207 (SE = 0.084) for the plasticity assay and 1.355 (SE = 0.097) for the common garden assay. All values indicate adequate model fit for the respective analyses.

Supplementary Table 13: Random temperature sequences. Weekly temperature sequences (°C) for the randomly fluctuating treatment, generated each Monday and applied to all three replicate populations. Each sequence cycled every 24 hours with 3-hour temperature steps.

Week	Temperature Sequence (°C)
1	30,24,27,33,27,24,21,30
2	30,27,33,27,21,30,24,24
3	33,27,21,24,27,24,30,30
4	24,27,24,33,30,21,30,27
5	24,21,27,27,30,30,24,33
6	27,30,24,24,33,30,27,21
7	30,27,33,21,30,27,24,24

8	24,30,21,27,30,24,33,27
9	27,21,24,33,30,27,24,30
10	33,24,30,24,30,27,27,21
11	24,21,27,30,27,30,33,24
12	27,24,33,30,30,21,24,27
13	21,24,30,24,33,27,30,27
14	21,27,27,24,30,30,24,33
15	33,27,27,30,30,21,24,24
16	27,30,24,24,27,21,30,33

Supplementary Figure 1. Raw survival data from the common garden (CG) assay. Boxplots showing the number of surviving flies across different experimental conditions and age intervals. Flies from three parent generations (Gen 4, 7, and 10) evolved in three different incubator temperature regimes (constant 27°C, predictably fluctuating, and randomly fluctuating) before being reared for two generations at 27°C. Data are binned into 10-day age intervals (0-9, 10-19, 20-29, etc. days) to aggregate survival counts that were originally measured twice weekly throughout the flies' lifespans. Each boxplot represents the distribution of surviving fly counts across four replicate vials (each initially containing two males and two females) for each treatment combination. The boxplots display the median (middle line), interquartile range (box), and range (whiskers), with outliers shown as individual points. These raw data complement the Cox proportional hazards model results presented in the main text (concordance = 0.696, SE = 0.007) by showing the actual observed survival patterns rather than modeled probabilities.

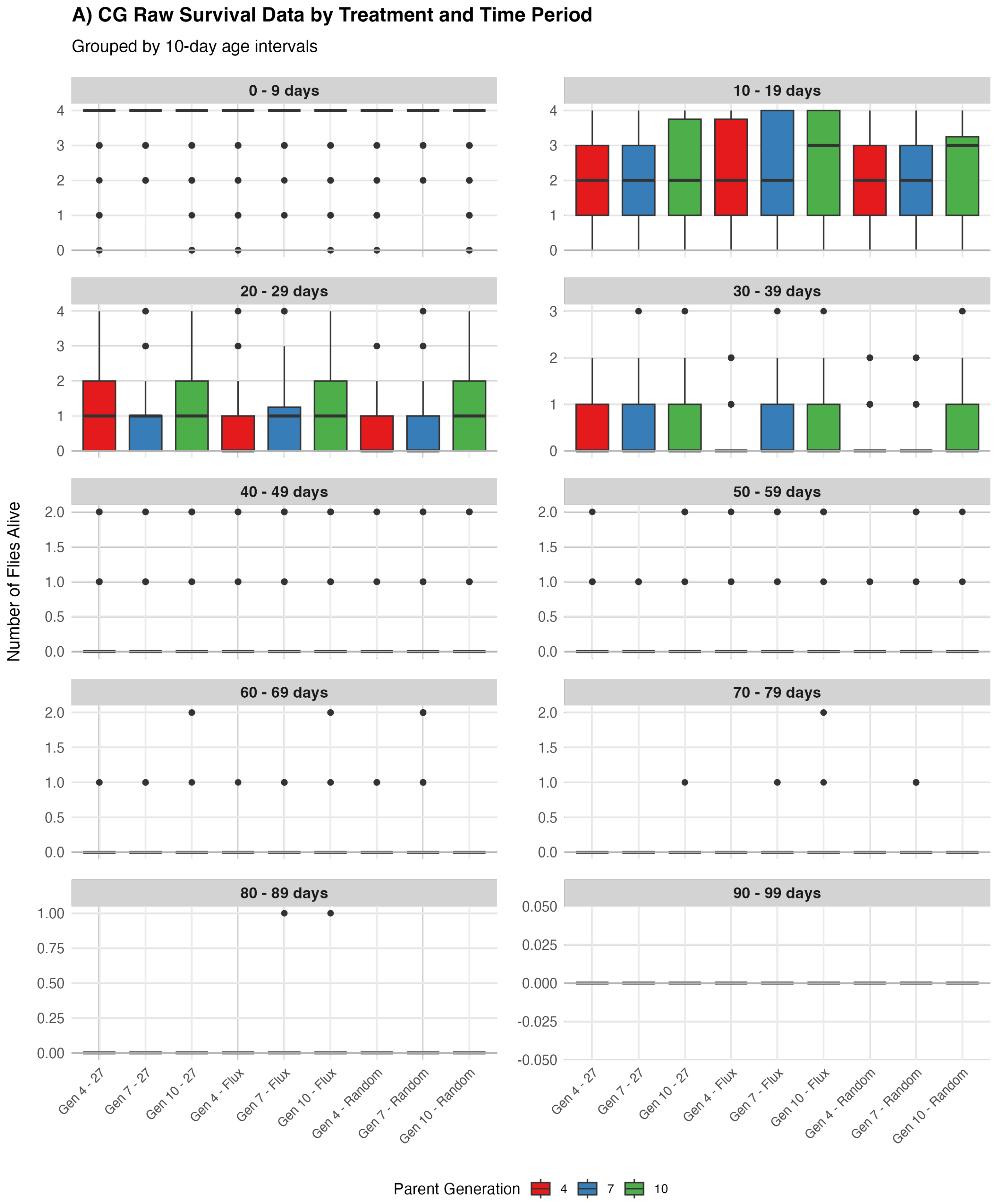
A) CG Raw Survival Data by Treatment and Time Period
Grouped by 10-day age intervals
0 - 9 days
10 - 19 days
20 - 29 days
30 - 39 days
40 - 49 days
50 - 59 days
60 - 69 days
70 - 79 days
80 - 89 days
90 - 99 days
Number of Flies Alive
Gen 4 - 27
Gen 7 - 27
Gen 10 - 27
Gen 4 - Flux
Gen 7 - Flux
Gen 10 - Flux
Gen 4 - Random
Gen 7 - Random
Gen 10 - Random
Parent Generation
4
7
10

Supplementary Figure 2: Raw survival data from the plasticity assay (PA). Boxplots showing the number of surviving flies across different experimental conditions and age intervals when directly tested in their respective temperature conditions. Flies from three parent generations (Gen 4, 7, and 10) that evolved in three different incubator temperature regimes (constant 27°C, predictably fluctuating, and randomly fluctuating) were tested immediately in response to test temperatures. Data are binned into 10-day age intervals (0-9, 10-19, 20-29, etc. days) from survival counts that were originally collected twice weekly. Each boxplot represents the distribution of surviving fly counts across four replicate vials (each initially containing two males and two females) for each treatment combination. Unlike the common garden experiment, these data reflect both developmental plasticity and genetic adaptation. These raw data supplement the Cox proportional hazards model results presented in the main text (concordance = 0.682, SE = 0.008) and show the immediate response patterns to temperature regimes without allowing for developmental acclimation.

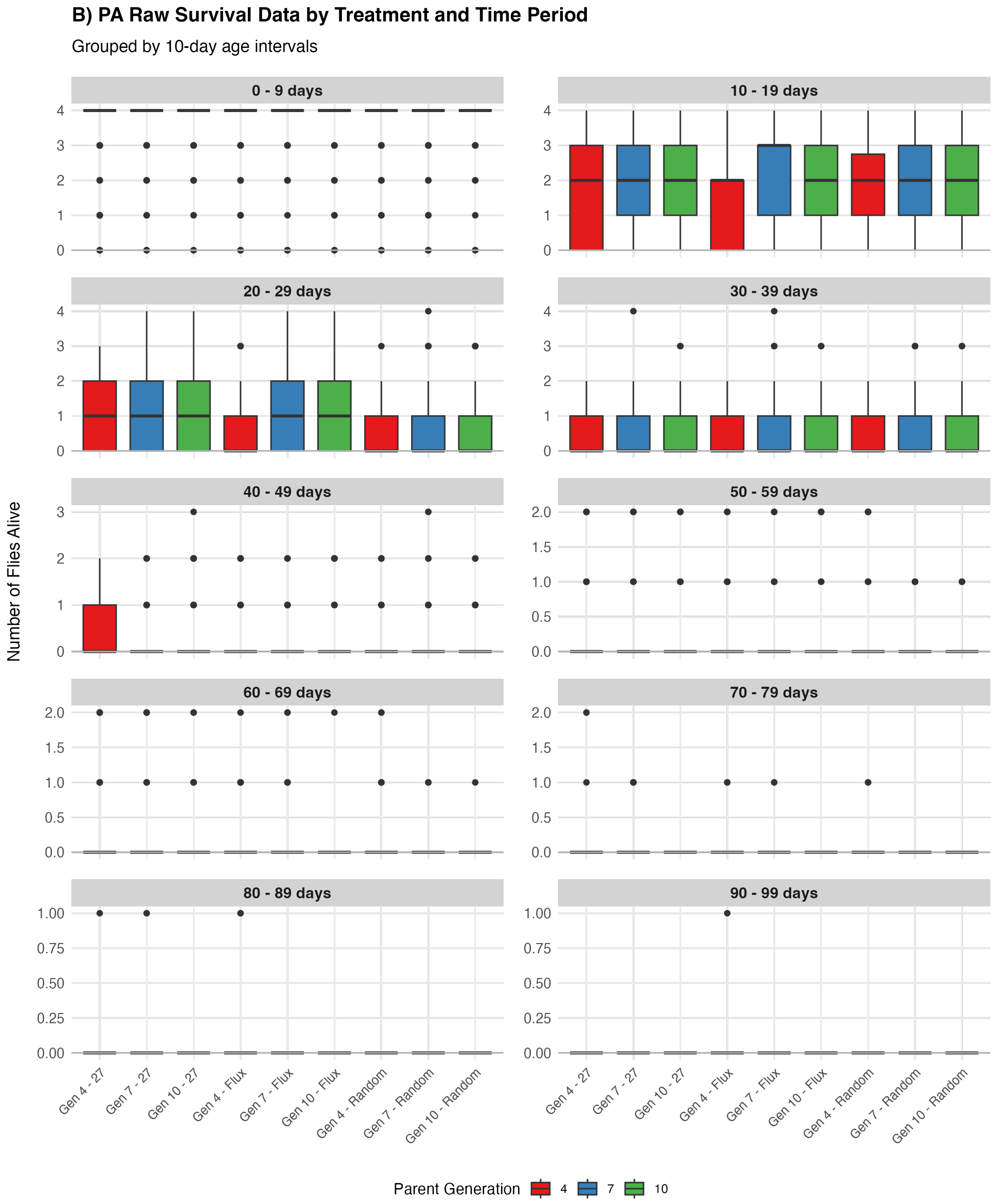
B) PA Raw Survival Data by Treatment and Time Period
Grouped by 10-day age intervals
0 - 9 days
10 - 19 days
20 - 29 days
30 - 39 days
40 - 49 days
50 - 59 days
60 - 69 days
70 - 79 days
80 - 89 days
90 - 99 days
Number of Flies Alive
Gen 4 - 27
Gen 7 - 27
Gen 10 - 27
Gen 4 - Flux
Gen 7 - Flux
Gen 10 - Flux
Gen 4 - Random
Gen 7 - Random
Gen 10 - Random
Parent Generation
4
7
10

Supplementary Figure 3: Raw egg production data across age, test temperature, and assay type. Points show the mean number of eggs per female across different ages and test temperatures (23°C to 31°C) for both the common garden (CG) and plasticity (PA) assays. Error bars represent standard error of the mean, and point size indicates the number of observations contributing to each mean. The raw data visualize the actual observations underlying the model predictions presented in the main text (Figures 3 and 5). These patterns confirm that higher temperatures generally reduce overall egg production and that egg production tends to decline with age across all temperatures in both assay types. The common garden assay (left panel) shows egg production patterns after two generations of standardized rearing, while the plasticity assay (right panel) reflects the immediate plastic response to different test temperatures.

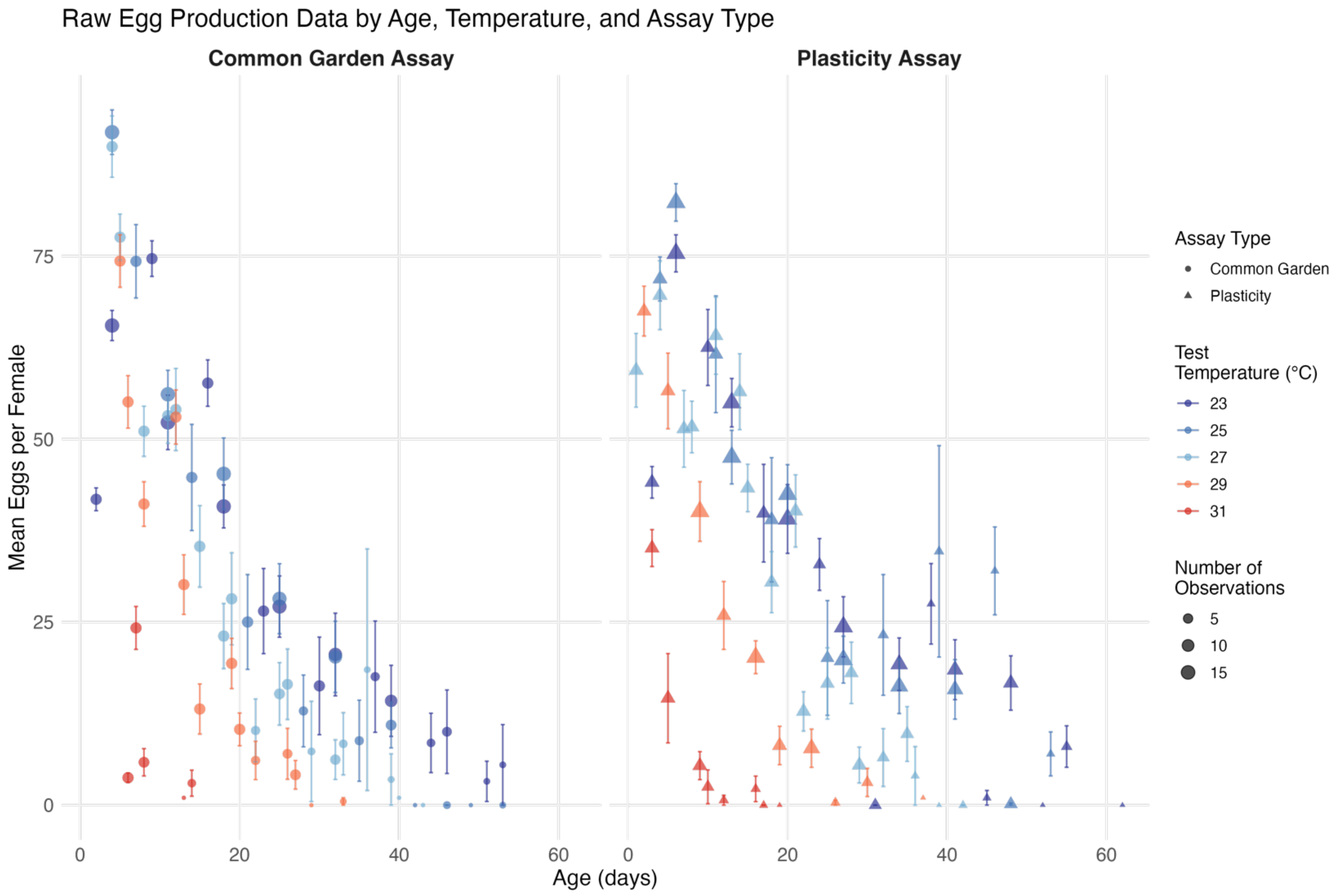

Supplementary Figure 4: Raw egg production data by parent generation, evolutionary environment, and assay type. Bars show the mean number of eggs per female for flies from parent generations 4 and 10 that evolved in three different evolutionary environments (constant 27°C, predictably fluctuating, and randomly fluctuating). The left panel shows data from the common garden assay (after two generations of standardized rearing at 27°C), while the right panel shows data from the plasticity assay (direct testing of flies from each evolutionary environment). Error bars represent 95% confidence intervals. These raw data complement the model-based predictions shown in Figures 3 and 5, revealing the actual observed differences in egg production between early and late generations across evolutionary treatments. The comparison between assay types highlights how the effects of evolutionary history on egg production patterns differ depending on whether flies experience a common rearing environment before testing.

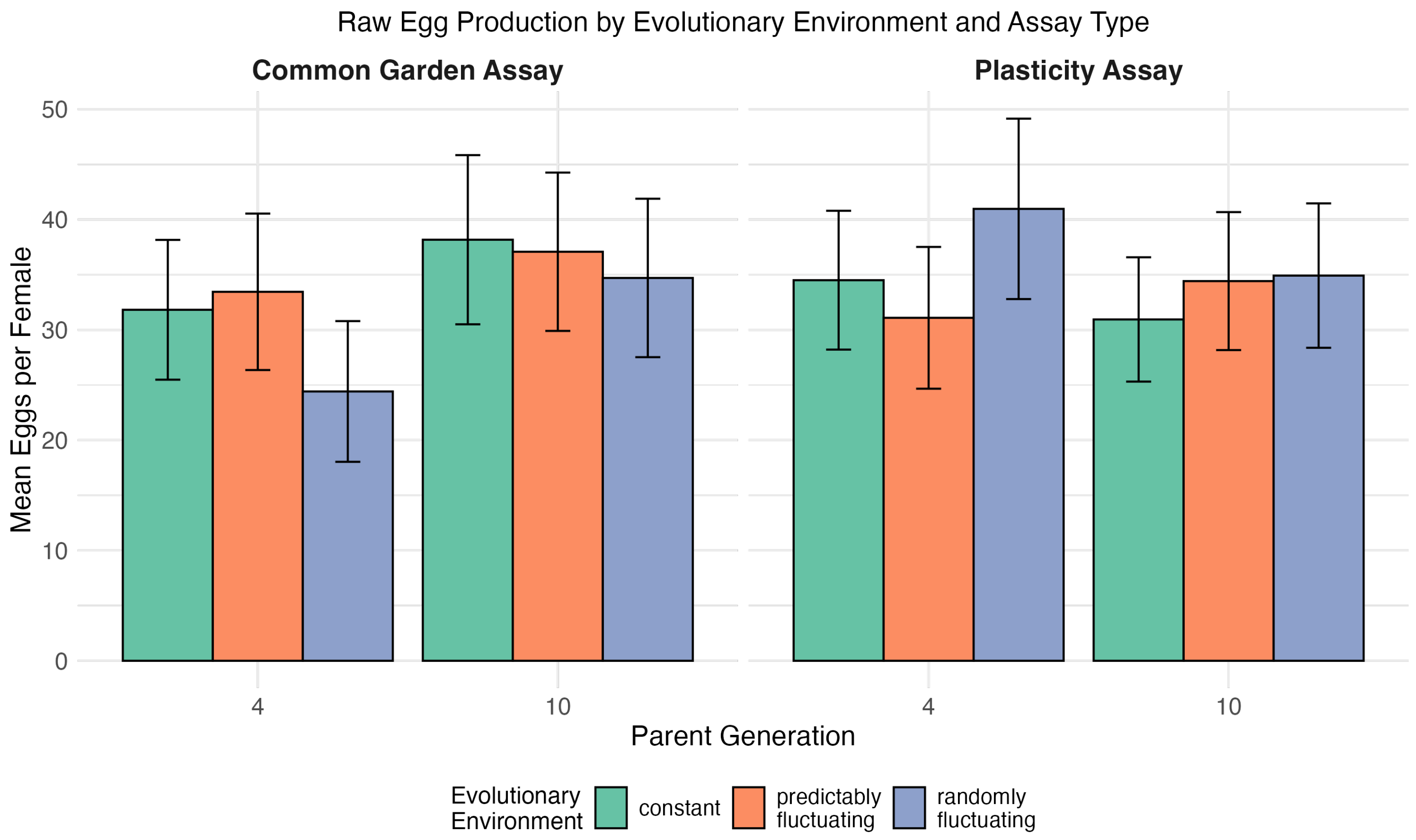

Supplement: qraf052_Supplemental_File [file qraf052_supplemental_file.pdf]
